# Comparison of the Prognostic Value of F-18 Pet Metabolic Parameters of Primary Tumors and Regional Lymph Nodes in Patients with Locally Advanced Cervical Cancer Who Are Treated with Concurrent Chemoradiotherapy

**DOI:** 10.1371/journal.pone.0137743

**Published:** 2015-09-14

**Authors:** Gun Oh Chong, Shin Young Jeong, Shin-Hyung Park, Yoon Hee Lee, Sang-Woo Lee, Dae Gy Hong, Jae-Chul Kim, Yoon Soon Lee, Young Lae Cho

**Affiliations:** 1 Department of Obstetrics and Gynecology, Kyungpook National University Medical Center/ School of Medicine, Daegu, Republic of Korea; 2 Department of Nuclear Medicine, Kyungpook National University Medical Center/ School of Medicine, Daegu, Republic of Korea; 3 Department of Radiation Oncology, Kyungpook National University Medical Center/School of Medicine, Daegu, Republic of Korea; University of California Davis, UNITED STATES

## Abstract

**Objective:**

This study investigated the metabolic parameters of primary tumors and regional lymph nodes, as measured by pre-treatment F-18 fluorodeoxyglucose positron emission tomography/computed tomography (F-18 FDG PET/CT) to compare the prognostic value for the prediction of tumor recurrence. This study also identified the most powerful parameter in patients with locally advanced cervical cancer treated with concurrent chemoradiotherapy.

**Methods:**

Fifty-six patients who were diagnosed with cervical cancer with pelvic and/or paraaortic lymph node metastasis were enrolled in this study. Metabolic parameters including the maximum standardized uptake value (SUVmax), the metabolic tumor volume (MTV), and total lesion glycolysis (TLG) of the primary tumors and lymph nodes were measured by pre-treatment F-18 FDG PET/CT. Univariate and multivariate analyses for disease-free survival (DFS) were performed using the clinical and metabolic parameters.

**Results:**

The metabolic parameters of the primary tumors were not associated with DFS. However, DFS was significantly longer in patients with low values of nodal metabolic parameters than in those with high values of nodal metabolic parameters. A univariate analysis revealed that nodal metabolic parameters (SUVmax, MTV and TLG), paraaortic lymph node metastasis, and post-treatment response correlated significantly with DFS. Among these parameters, nodal SUVmax (hazard ratio [HR], 4.158; 95% confidence interval [CI], 1.1–22.7; *p* = 0.041) and post-treatment response (HR, 7.162; 95% CI, 1.5–11.3; *p* = 0.007) were found to be determinants of DFS according to a multivariate analysis. Only nodal SUVmax was an independent pre-treatment prognostic factor for DFS, and the optimal cutoff for nodal SUVmax to predict progression was 4.7.

**Conclusion:**

Nodal SUVmax according to pre-treatment F-18 FDG PET/CT may be a prognostic biomarker for the prediction of disease recurrence in patients with locally advanced cervical cancer.

## Introduction

Cervical cancer is the third most commonly diagnosed cancer and the fourth leading cause of cancer-related death in women [1]. Advanced International Federation of Gynecology and Obstetrics (FIGO) stage, larger tumor size, and presence of lymph node metastasis have been reported as negative prognostic factors for patients with cervical cancer who are treated with concurrent chemoradiotherapy (CCRT) [2,3]. Pelvic and/or paraaortic lymph node status is a strong independent prognostic factor in cervical cancer [4,5]. Lymph node metastases in cervical cancer have the propensity to progress in an orderly fashion from the pelvis to the abdomen, the supraclavicular region, and then to the mediastinum. For patients with locally advanced cervical cancer (stage IIB to IV), CCRT with a cisplatin-based regimen has become the standard treatment [6,7]. Despite advances in treatment, a substantial fraction of patients do not respond to therapy and continue to face a dismal prognosis.

Currently, F-18 fluorodeoxyglucose positron emission tomography/computed tomography (F-18 FDG PET/CT) has been widely used to detect lymph node involvement, distant metastasis, and recurrent disease in patients with cervical cancer [8]. Previous studies have demonstrated that the maximum standardized uptake value (SUVmax) and metabolic parameters of the primary tumor correlated with lymph node metastasis, persistent disease after treatment, pelvic disease recurrence, and poor overall survival (OS) [9,10]. Moreover, recent studies have reported that a high SUVmax of the regional lymph nodes is a significant adverse factor in patients with cervical cancer [11–13]. In regards to its prognostic value, however, the degree of F-18 FDG uptake in regional lymph nodes on F-18 FDG PET/CT has not been fully investigated in patients with locally advanced cervical cancer. In addition, no comparison study has been performed on the prognostic value of F-18 FDG uptake in primary tumors and regional lymph nodes in patients with locally advanced cervical cancer who had regional lymph node involvement as diagnosed by F-18 FDG PET/CT. Moreover, the prognostic value of the nodal metabolic tumor volume (MTV) and total lesion glycolysis (TLG) has not been studied in cervical cancer.

The aim of the present study was to compare the prognostic value of metabolic parameters including SUVmax, MTV and TLG of primary tumors and regional lymph nodes to predict tumor recurrence and to identify the most powerful biological marker that can be measured by pre-treatment F-18 FDG PET/CT in patients with locally advanced cervical cancer who are treated with CCRT. In addition, we evaluated the relationship between nodal F-18 FDG uptake and known prognostic parameters of cervical cancer.

## Materials and Methods

### Patients

For this study, we enrolled 95 patients with biopsy-proven cervical cancer, which was treated with CCRT between September 2005 and August 2014. Retrospective data collection and analysis were approved by the Institutional Review Board of Kyungpook National University Medical Center. The need for informed consent was waived due to the retrospective design of the study. The patients were staged according to the FIGO staging system. All patients had undergone F-18 FDG PET/CT for the initial diagnosis and staging, as well as for the planning of the radiotherapy. Of these patients, 56 patients were confirmed to have pelvic and/or paraaortic lymph node involvement without distant metastasis on F-18 FDG PET/CT. We then performed a retrospective review of 56 patients with locally advanced cervical cancer. The following clinical and pathological parameters were retrieved and reviewed: age, FIGO stage, histology, primary tumor size, and paraaortic lymph node metastasis.

### Treatment

All patients were treated with a combination of external beam radiotherapy (EBRT) and high-dose-rate (HDR) intracavitary brachytherapy (ICR) with curative intent. EBRT was delivered to the whole pelvis using 10 MV photons with customized shielding in 1.8 Gy daily fractions, five times a week, for a total dose of 45 Gy. A four-field box technique was used. The superior border was the L4–L5 vertebral level. The inferior border was at the bottom of the obturator foramen or 2–3 cm below the lowest extent of the cervical or vaginal disease. The lateral borders were placed 2 cm lateral to the inner bony margins of the true pelvis. For the lateral fields, the anterior border included the symphysis pubis, and the posterior border was the S2-3 interspace. For patients with paraaortic nodal involvement, the superior border extended to the T12-L1 interspace. A boost of EBRT given in 5 fractions for a total of 10 Gy was indicated for patients with parametrial involvement and/or nodal metastases. HDR ICR was initiated after an EBRT dose of 39.6 Gy. ICR was delivered twice a week in five fractions with a fractional dose of 6 Gy at point A. At the end of parametrial and nodal boost EBRT, F-18 FDG PET/CT or CT scans were performed. In cases of residual pelvic lymphadenopathy, an additional boost of EBRT, which consisted of 4–10 Gy, was applied. As a result, a median of 65 Gy (range, 59–65 Gy) of radiation was irradiated for gross residual lymphadenopathy after parametrial and nodal boost. Weekly cisplatin at a dose of 40 mg/m^2^ was administered during radiotherapy. The first course of cisplatin was administered on day 1 of radiotherapy.

### F-18 FDG PET/CT Image Acquisition

All patients fasted for at least 6 hours, and their blood glucose levels were determined before the administration of F-18 FDG. Patients with blood glucose levels higher than 150 mg/dL were rescheduled for a later examination, and treatment was administered to maintain a blood glucose concentration of <150 mg/dL in all subjects. Patients received intravenous injections of approximately 8.1 MBq of FDG per kg of body weight and were advised to rest for 1 hour before the acquisition of the F-18 FDG PET/CT image. F-18 FDG PET/CT scans were performed using a Reveal RT-HiREZ 6-slice CT apparatus (CTI Molecular Imaging, Knoxville, TN, USA) and a 16-slice CT Discovery STE apparatus (GE Healthcare, Milwaukee, WI, USA). Before the PET scan, for attenuation correction, a low-dose CT scan was obtained without contrast enhancement from the skull base to the thigh when the patient was supine and breathing quietly. PET scans with a maximum spatial resolution of 6.5 mm (Reveal PET/CT) and 5.5 mm (Discovery PET/CT) were also obtained from the skull base to the thigh at 3 minutes per bed position. PET images that were obtained with the Reveal PET/CT and Discovery PET/CT scanners were reconstructed with a 128 × 128 matrix, an ordered-subset expectation maximum iterative reconstruction algorithm (4 iterations; 8 subsets), a Gaussian filter of 5.0 mm, and a slice thickness of either 3.0 mm (Reveal PET/CT) or 3.27 mm (Discovery PET/CT).

### Image analysis

The display and the analysis of the images were achieved with Volume Viewer software on an Advantage Workstation 4.5 (GE Medical Systems, Milwaukee, WI, USA), which provides a convenient and automatic method to delineate the volume of interest using an isocontour threshold method based on the SUV. For each patient, the SUVmax was designated as the highest SUVmax of the primary tumor and regional lymph nodes, and MTV and TLG were obtained by summation of the values of the primary tumor and the values of all of the regional lymph nodes. The SUVmax was obtained with the following formula: SUVmax = maximum activity in the region of interest (MBq/g)/(injected dose [MBq]/body weight [g]). The mean SUV of the mediastinal background plus two standard deviations was used as the threshold to automatically calculate MTV. The TLG of a lesion was calculated as the product of MTV and the SUVmean. The MTV and TLG of the regional lymph nodes were defined as the sum of MTV and TLG values of each lymph node. Semiquantitative and volumetric analyses of the primary tumor were performed using PETVCAR (PET Volume Computerized Assisted Reporting) on an Advantage Workstation 4.3 (GE Healthcare, Milwaukee, WI, USA).

Metabolic responses were evaluated by nuclear medicine physicians during and after CCRT using the F-18 FDG PET/CT images. A complete response (CR) was defined as the absence of abnormal F-18 FDG uptake in the primary cervical tumor and the absence of abnormal uptake by the metastatic lymph nodes, as noted in the pretreatment F-18 FDG PET/CT. Partial response (PR) was defined as any persistent abnormal uptake at these sites. Progressive disease (PD) was defined as new foci of abnormal F-18 FDG uptake, which is suggestive of metastasis.

### Clinical endpoints and follow-up

Clinical follow-up of patients was performed every 3 months for 2 years, and then every 6 months from 2 to 5 years, and annually thereafter. After the completion of treatment, F-18 FDG PET/CT was performed in all patients. Failure was defined as biopsy-proven recurrence or documentation of disease progression based on serial imaging studies. Failure patterns were divided into the following four groups: none, isolated local failure that included the central pelvis and/or pelvic lymph nodes, distant failure that included paraaortic and supraclavicular lymph nodes, and combined local and distant failure. To evaluate the prognostic value of the clinical and quantitative metabolic parameters, DFS was chosen as an endpoint. DFS was calculated from the date of diagnosis of disease to the date of diagnosis of recurrence or the date of the last follow-up.

### Statistical analysis

The time to event was calculated as the time interval from the date of diagnosis to the date of the first clinical or imaging findings that suggested disease recurrence. The differences between subsets were evaluated by Student’s t-test, and differences between proportions were compared with the chi square test. To identify an optimal cutoff of the F-18 FDG uptake values of the primary tumor and regional lymph nodes for the prediction of recurrence, receiver operating characteristics (ROC) curve analysis was performed. The Kaplan-Meier method and the log-rank test were used in the survival analysis of prognostic factors. The Cox proportional hazard model was used to evaluate prognostic variables for univariate and multivariate comparisons, and an estimated hazard ratio (HR) with 95% confidence intervals (95% CI) is presented. Statistical analyses were performed with SPSS 21.0 for Windows (IBM Corporation, Armonk, NY, USA), and *p*-values <0.05 were considered statistically significant.

## Results

### Clinical features and treatment outcomes

The clinical characteristics of the study participants are listed in Table 1. The predominant FIGO stage was IIB (44 patients), followed by IIIA2 (5 patients), IIIB (5 patients), and IIIA1 (3.6%). Thirteen patients (23.2%) had paraaortic nodal metastases. The SUVmax, MTV, and TLG of the primary tumors were 16.9 ± 9.3, 99.0 ± 116.8 cm^3^, and 753.9 ± 971.9, respectively. The SUVmax, MTV, and TLG of the lymph nodes were 7.5 ± 7.1, 16.0 ± 33.5 cm^3^, and 72.5 ± 179.2, respectively. F-18 FDG PET/CT images were obtained for all patients both before and after treatment. Post-treatment F-18 FDG PET/CT images were obtained within a median of 5 months (range, 2–18 months) after the completion of CCRT. According to F-18 FDG PET/CT performed after treatment, complete response (CR), partial response (PR) and progressive disease (PD) were observed in 37 patients (66.1%), 17 patients (30.4%), and 2 patients (3.6%), respectively. After the median follow-up of 42 months (range, 6–97 months), 18 patients (32.1%) had recurrence, and 11 patients (19.6%) had died due to disease progression. Of the 18 patients who experienced disease recurrence, 5 patients had local recurrence only, 5 patients had distant recurrence only, and 8 patients had both local and distant recurrence (Table 2).

**Table 1:** Distribution of the clinical characteristics and metabolic parameters according to regional lymph node SUVmax (high and low).

Variable	All patients (N = 56)	Nodal SUVmax ≤4.7 (N = 25)	Nodal SUVmax >4.7 (N = 31)	*P*
Age (years)	51.5 ± 10.3	53.2 ± 11.3	50.1 ± 10.4	0.286
FIGO stage (N, %)				0.041
IIB	44 (78.6%)	22 (88.0%)	22 (71.0%)	
IIIA1	2 (3.6%)	2 (8.0%)	0 (0%)	
IIIA2	5 (8.9%)	1 (4.0%)	4 (12.9%)	
IIIB	5 (8.9%)	0 (0%)	5 (16.1%)	
Histology (N, %)				0.020
Squamous cell carcinoma	50 (89.3%)	25 (100.0%)	25 (80.6%)	
Adenocarcinoma	6 (10.7%)	0 (0%)	6 (19.4%)	
Primary tumor size (cm)	4.6 ± 1.7	4.1 ± 1.2	5.1 ± 1.9	0.045
Paraaortic lymph node metastasis (N, %)	13 (23.2%)	4 (16.0%)	9 (29.0%)	0.251
Primary tumor SUVmax	16.9 ± 9.3	16.9 ± 9.5	16.8 ± 9.3	0.954
Primary tumor MTV	99.0 ± 116.8	66.5 ± 51.0	125.2 ± 146.1	0.044
Primary tumor TLG	753.9 ± 971.9	516.26 ± 564.5	945.5 ± 1179.7	0.081
Nodal SUVmax	7.5 ± 7.1	3.4 ± 0.7	10.9 ± 8.2	<0.001
Nodal MTV	16.0 ± 33.5	2.6 ± 2.6	26.8 ± 42.2	0.003
Nodal TLG	72.5 ± 179.2	6.8 ± 5.8	125.9 ± 222.8	0.007

FIGO = International Federation of Gynecology and Obstetrics; SUVmax = maximum standardized uptake value; MTV = whole-body metabolic tumor volume; TLG = total lesion glycolysis

**Table 2:** Treatment response and recurrence patterns according to the SUVmax of the regional lymph nodes (high and low).

Variables	All patients (N = 56)	Nodal SUVmax ≤4.7 (N = 25)	Nodal SUVmax >4.7 (N = 37)	*P*
Response (N, %)				0.032
Complete response	37 (66.1%)	21 (84.0%)	16 (51.6%)	
Partial response	17 (30.4%)	4 (16.0%)	13 (41.9%)	
Progressive disease	2 (3.6%)	0 (0%)	2 (6.5%)	
Recurrence (N, %)				0.002
Local	5 (8.9%)	2 (8.0%)	3 (9.7%)	
Distant	5 (8.9%)	0 (0%)	5 (16.1%)	
Combined	8 (14.3%)	0 (0%)	8 (25.8%)	

SUVmax = maximum standardized uptake

### Cut-off value of metabolic parameters

**Fig 1:**
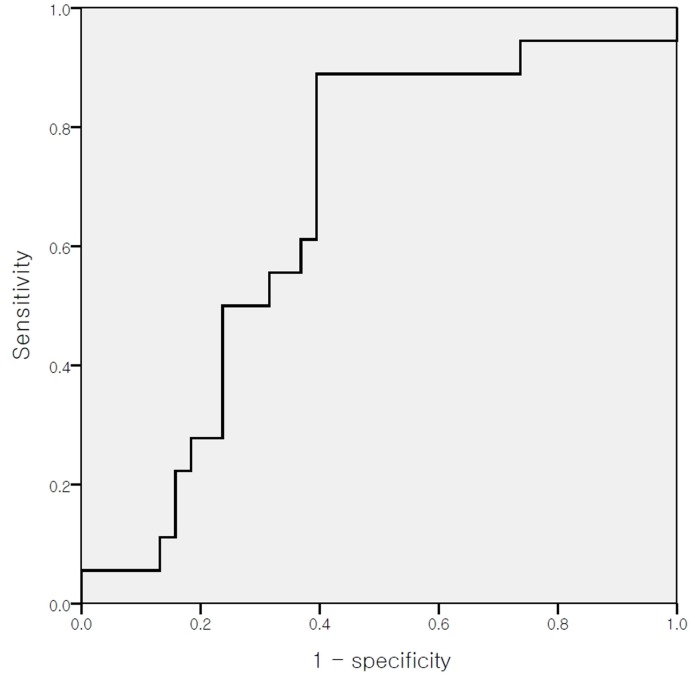
Receiver operating characteristic curve analysis for the prediction of recurrence according to the nodal maximum standardized uptake value (SUVmax). The area under the curve was 0.668 (*p* = 0.044, 95% confidence interval 0.519–0.817), and 4.7 was determined to be the cutoff comparison value for the nodal SUVmax.

The optimal cut-off values for the SUVmax, MTV, and TLG, which were calculated using the ROC curve for the primary tumor, were 12.3, 63.7 cm^3^, and 411.9, respectively (*p* = 0.158 for SUVmax, 95% CI 0.237–0.528; *p* = 0.086 for MTV, 95% CI 0.360–0.697; and *p* = 0.084 for TLG, 95% CI 0.334–0.663). The optimal cut-off values that were calculated for the nodal SUVmax, MTV, and TLG were 4.7, 10.3 cm^3^, and 32.9, respectively (*p* = 0.044 for SUVmax, 95% CI 0.519–0.817; *p* = 0.046 for MTV, 95% CI 0.509–0.824; and *p* = 0.038 for TLG, 95% CI 0.519–0.826) (Fig 1).

### Survival Analyses

**Fig 2:**
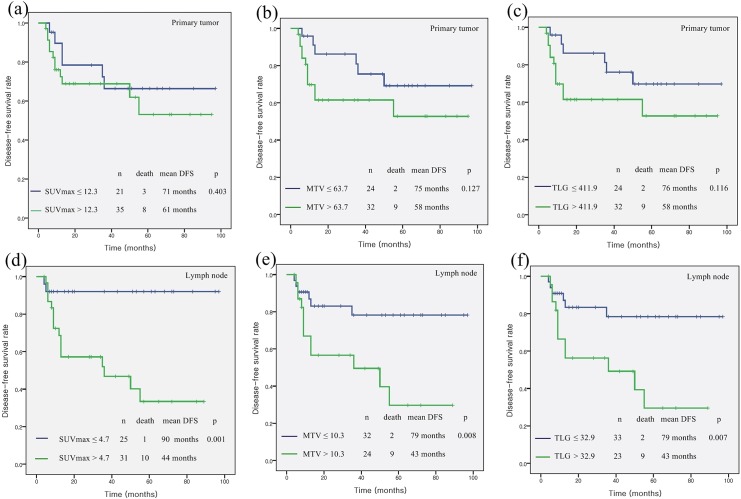
Kaplan-Meier survival plots of disease-free survival according to the metabolic PET parameters. Kaplan-Meier survival plots of disease-free survival according to (a) the maximum standardized uptake value (SUVmax) of the primary tumor, (b) the metabolic tumor volume (MTV) of the primary tumor, (c) total lesion glycolysis (TLG) of the primary tumor, (d) the SUVmax of the regional lymph nodes, (e) the MTV of the regional lymph nodes, and (f) TLG of the regional lymph nodes.

No differences in DFS were observed according to the F-18 FDG uptake in the primary tumors. The estimated five-year DFS rates were 66% in patients with a low SUVmax of the primary tumor (≤12.3) and 53% in patients with a high SUVmax of the primary tumor (>12.3; *p* = 0.403). The estimated five-year DFS rates were 69% in patients with a low MTV of the primary tumor (≤63.7 cm^3^) and a low TLG (≤411.9) of the primary tumor, compared with 53% in patients with a high MTV of the primary tumor (>63.7 cm3; *p* = 0.127) and a high TLG of the primary tumor (>411.9; *p* = 0.116). However, the estimated five-year DFS rate was significantly lower in patients with a high nodal F-18 FDG uptake compared with those with a low nodal F-18 FDG uptake (nodal SUVmax ≤4.7 vs. >4.7, 92% vs. 33%, *p* = 0.001; nodal MTV ≤10.3 cm^3^ vs. >10.3 cm^3^, 78% vs. 30%, *p* = 0.008; and nodal TLG ≤32.9 vs. >32.9, 78% vs. 30%, *p* = 0.007, respectively). Kaplan-Meier survival plots showed significant differences in DFS when the data were stratified by the nodal SUVmax, the MTV, and TLG (Fig 2).

According to the univariate analysis, the nodal SUVmax (HR, 7.592; 95% CI, 1.8–34.8; *p* = 0.006), the nodal MTV (HR, 6.015; 95% CI, 1.3–9.2; *p* = 0.014), the nodal TLG (HR, 6.266; 95% CI, 1.3–9.4; *p* = 0.012), paraaortic lymph node metastasis (HR, 5.393; 95% CI, 1.2–8.5; *p* = 0.020), and post-treatment response (HR, 13.289; 95% CI, 2.4–17.0; *p* < 0.001) were significant prognostic factors for DFS. With the use of the forward stepwise multivariate Cox proportional hazards model, the nodal SUVmax (HR, 4.158; 95% CI, 1.1–22.7; *p* = 0.041) and the post-treatment response (HR, 7.162; 95% CI, 1.5–11.3; *p* = 0.007) remained significant prognostic factors for DFS (Table 3).

**Table 3:** Univariate and multivariate analyses of clinical variables and quantitative metabolic parameters for disease-free survival.

Variables	Univariate analysis			Multivariate analysis		
	HR	95% CI	*P*	HR	95% CI	*P*
Age (years) <45 vs. ≥45	1.030	0.6–4.2	0.310			
Primary tumor SUVmax >12.3 vs. ≤12.3	0.673	0.5–4.0	0.412			
Primary tumor MTV >63.7 cm^3^ vs. ≤63.7 cm^3^	2.172	0.8–5.6	0.141			
Primary tumor TLG >411.9 vs. ≤411.9	2.294	0.8–5.8	0.130			
Nodal SUVmax >4.7 vs. ≤4.7	7.592	1.8–34.8	0.006	4.158	1.1–22.7	0.041
Nodal MTV >10.3 cm^3^ vs. ≤10.3 cm^3^	6.015	1.3–9.2	0.014			
Nodal TLG >32.9 vs. ≤32.9	6.266	1.3–9.4	0.012			
FIGO stage >IIB vs. IIB	1.695	0.7–5.2	0.193			
Tumor size ≥4 cm vs. <4 cm	0.811	0.5–5.1	0.368			
Paraaortic node metastases	5.393	1.2–8.5	0.020			
Post-treatment response PR or PD vs. CR	13.289	2.4–17.0	<0.001	7.162	1.5–11.3	0.007

SUVmax = maximum standardized uptake value; MTV = whole-body metabolic tumor volume; TLG = total lesion glycolysis; FIGO = International Federation of Gynecology and Obstetrics; CR = complete response; PR = partial response; PD = progressive disease; HR = hazard ratio; CI = confidence interval.

### Correlation between the nodal SUVmax and clinicopathological parameters

High nodal SUVmax (>4.7) was associated with FIGO stage (*p* = 0.041), histology (*p* = 0.020), primary tumor size (*p* = 0.045), and primary tumor MTV (*p* = 0.044). No correlation was found between the primary tumor SUVmax and the nodal SUVmax (*p* = 0.954) (Table 1). In the low nodal SUVmax (≤4.7) group, 21 patients (84.0%) had a CR and 4 patients (16.0%) had a PR. In the high nodal SUVmax group (>4.7), 16 patients (51.6%) had a CR, 13 patients (41.9%) had a PR, and 2 patients (6.5%) had PD (*p* = 0.032). In the low nodal SUVmax group, only local recurrences were observed. In the high nodal SUVmax group, however, both distant (16.1%) and combined recurrences (25.8%) were observed (*p* = 0.004) (Table 2).

## Discussion

This study investigated the relationship between quantitative metabolic parameters and DFS in patients with locally advanced cervical cancer with regional lymph node involvement who were treated by CCRT. To our knowledge, this is the first study that suggests that nodal metabolic parameters as measured on F-18 FDG PET/CT are more significant prognostic parameters compared with metabolic parameters of the primary tumor in cases of regional lymph node metastasis that are confirmed by F-18 FDG PET/CT. Among the metabolic parameters of the primary tumor and the lymph nodes, the nodal SUVmax was the most powerful prognostic marker for the prediction of recurrence.

Stage, tumor size, and lymph node status have been recognized as important predictors of the prognosis with respect to patient survival [2,3]. In addition, it has been well demonstrated that the survival rate of patients with cervical cancer with lymph node metastasis is significantly lower than that of patients without lymph node metastasis [14,15]. However, an accurate lymph node status can be determined only after surgery, which is frequently associated with significant morbidity and mortality. F-18 FDG PET/CT has been shown to be more sensitive than CT or magnetic resonance imaging for the detection of lymph node metastasis in patients with cervical cancer [16,17]. Moreover, F-18 FDG uptake reflects the degree of glucose metabolism within the tumor, which represents the aggressiveness of the malignant lesion [18,19].

Several reports have suggested that locally advanced cervical cancers in which the primary tumors show high F-18 FDG uptake are associated with a poorer prognosis than cervical cancers with low uptake [10,20]. Recently, a few studies have demonstrated that regional lymph node F-18 FDG uptake might be a prognostic biomarker in patients with cervical cancer who are treated with CCRT [11–13]. Yen et al. [11] found that an SUVmax >3.3 in the paraaortic lymph nodes was predictive of worse OS; however, they found no impact of the pelvic lymph node SUVmax on OS. In another study, Kidd et al. [12] showed that an SUV ≥4.3 in the pelvic lymph nodes was a prognostic biomarker that was predictive of treatment response, pelvic recurrence risk, and DFS in 83 patients with cervical cancer and pelvic node involvement on PET/CT. Moreover, a recent study demonstrated that high F-18 FDG uptake in the pelvic lymph nodes was associated with a high risk of disease recurrence and poor survival in 93 of 183 patients with pelvic and/or paraaortic lymph node metastasis. In this earlier study, cervical cancer patients with a relatively early FIGO stage were enrolled. Fifty-four (77.1%) patients with FIGO stage I-II were enrolled in the study by Yen et al. [11], while 19 patients (22.9%) with FIGO stage IB1-IIA were included in the study by Kidd et al. [12]; 11 patients (11.8%) with FIGO stage IB2-IIA were described in the study by Onal et al. [13]. In the current study, however, the enrolled patients were restricted to those with locally advanced cervical cancer (≥FIGO stage IIB) who underwent pre-treatment F-18 FDG PET/CT that revealed pelvic and/or paraaortic lymph node metastasis. Moreover, we evaluated the F-18 FDG uptake values of whole regional lymph nodes including the pelvic and paraaortic lymph nodes. High values of metabolic parameters such as MTV and TLG of the whole regional lymph nodes have significant predictive value in terms of DFS. Moreover, the nodal SUVmax, including the SUVmax of the pelvic and paraaortic lymph nodes, was the most powerful prognostic marker for the prediction of recurrence among the metabolic parameters that were measured by pre-treatment F-18 FDG PET/CT. Furthermore, an elevated nodal SUVmax was also found to be predictive of worse OS (*p* = 0.016). However, the SUVmax of the primary tumor was not found to be correlated with OS (*p* = 0.233). The Kaplan-Meier curves of OS indicated better outcomes for patients with a lower SUVmax of the regional lymph nodes (S1 Fig).

Previously, we reported that the nodal SUVmax according to pretreatment F-18 FDG PET/CT might be an independent prognostic factor for disease recurrence in patients with breast cancer [21]. The hypothesis for the predictive value of the nodal SUVmax may be applied to cervical cancer in the same manner. First, many authors have reported that increased tumoral uptake of F-18 FDG correlates closely with the density of viable carcinoma cells, microvessel density, and proliferative activity [22,23]. Second, lymph node involvement has been known to be one of the most important prognostic factors in cervical cancer. We postulated that these combinatorial effects might highlight the prognostic significance of the nodal SUVmax.

F-18 FDG PET/CT can be used to evaluate treatment response. Several studies have shown that the metabolic response is associated with long-term outcomes in cervical cancer [24,25]. In this study, the post-treatment metabolic response was an independent prognostic factor for the prediction of disease recurrence in a multivariate analysis. However, the time frame for when F-18 FDG PET/CT was conducted after the completion of treatment, was varied (range, 2–18 months). As a result, the effect of various time intervals may limit the generalizability of our results.

Our study has some limitations. First, it is a retrospective study with a limited number of patients. Second, the SUV of small metastatic lymph nodes may be underestimated because of partial-volume effects and because of the limited resolution of PET [26]. Third, histopathological verification of the lymph nodes was not performed. The two types of PET scans that were used for this study might affect the values of the quantitative metabolic parameters as measured by FDG PET/CT. However, we performed image analysis on a single workstation to minimize the effects of the SUV measured from different scanners. Finally, the fact that only node-positive patients were enrolled may limit the generalizability of our results and prevent the application of risk stratification to patients without lymph node metastasis.

Despite these limitations, our study offers some unique and significant findings, and it differs from previous studies. We enrolled only patients with locally advanced cervical cancer who had higher than FIGO stage IIB disease. To our knowledge, this is the first study that compared the prognostic value of metabolic parameters of primary tumors and lymph nodes in patients with cervical cancer who had F-18 FDG PET/CT-proven pelvic and/or paraaortic nodal metastasis. In cases of nodal metastasis, the metabolic parameters of the primary tumor were not significant prognostic factors for tumor recurrence. Furthermore, conventional clinical factors for recurrence, such as age, size of the primary tumor, and FIGO stage, were not significantly predictive of DFS according to a univariate analysis. However, nodal metabolic parameters were significant predictors of DFS. In addition, the nodal SUVmax was the only predictive factor for DFS among the pre-treatment variables according to a multivariate analysis. Additionally, the optimal cutoff for the nodal SUVmax to differentiate progression was 4.7.

## Conclusion

The present study revealed that the nodal SUVmax on F-18 FDG PET/CT before CCRT may be an independent prognostic factor for the prediction of disease recurrence in patients with locally advanced cervical cancer with regional lymph node involvement. These findings may be used to improve prognostic models with appropriate risk reduction strategies. The identification of these high-risk patients may make it possible to personalize treatment that may involve higher doses of radiation boosts, consolidation chemotherapy, or adjuvant hysterectomy, when indicated.

## Supporting Information

S1 Fig Kaplan-Meier survival plots of overall survival according to metabolic PET parameters. Kaplan-Meier survival plots of disease-free survival according to (a) the primary tumor maximum standardized uptake value (SUVmax), (b) the nodal SUVmax.
(EPS)

## References

[1] Jemal A, Bray F, Center MM, Ferlay J, Ward E, Forman D. Global cancer statistics. CA Cancer J Clin. 2011;61(2):69–90. 10.3322/caac.20107 21296855

[2] Lee DW, Kim YT, Kim JH, Kim S, Kim SW, Nam EJ, et al Clinical significance of tumor volume and lymph node involvement assessed by MRI in stage IIB cervical cancer patients treated with concurrent chemoradiation therapy. J Gynecol Oncol. 2010;21(1):18–23. 10.3802/jgo.2010.21.1.18 20379443 PMC2849943

[3] Endo D, Todo Y, Okamoto K, Minobe S, Kato H, Nishiyama N. Prognostic factors for patients with cervical cancer treated with concurrent chemoradiotherapy: a retrospective analysis in a Japanese cohort. J Gynecol Oncol. 2015;26(1):12–8. 10.3802/jgo.2015.26.1.12 25310853 PMC4302279

[4] Kim SM, Choi HS, Byun JS. Overall 5-year survival rate and prognostic factors in patients with stage IB and IIA cervical cancer treated by radical hysterectomy and pelvic lymph node dissection. Int J Gynecol Cancer. 2000;10(4):305–12. 11240691 10.1046/j.1525-1438.2000.010004305.x

[5] Samlal RA, van der Velden J, Ten Kate FJ, Schilthuis MS, Hart AA, Lammes FB. Surgical pathologic factors that predict recurrence in stage IB and IIA cervical carcinoma patients with negative pelvic lymph nodes. Cancer. 1997;80(7):1234–40. 9317173

[6] Green JA, Kirwan JM, Tierney JF, Symonds P, Fresco L, Collingwood M, et al Survival and recurrence after concomitant chemotherapy and radiotherapy for cancer of the uterine cervix: a systematic review and meta-analysis. Lancet. 2001;358(9284):781–6. 11564482 10.1016/S0140-6736(01)05965-7

[7] Whitney CW, Sause W, Bundy BN, Malfetano JH, Hannigan EV, Fowler WC Jr, et al Randomized comparison of fluorouracil plus cisplatin versus hydroxyurea as an adjunct to radiation therapy in stage IIB-IVA carcinoma of the cervix with negative para-aortic lymph nodes: a Gynecologic Oncology Group and Southwest Oncology Group study. J Clin Oncol. 1999;17(5):1339–48. 10334517 10.1200/JCO.1999.17.5.1339

[8] Wong TZ, Jones EL, Coleman RE. Positron emission tomography with 2-deoxy-2-[(18)F]fluoro-D-glucose for evaluating local and distant disease in patients with cervical cancer. Mol Imaging Biol. 2004;6(1):55–62. 15018829 10.1016/j.mibio.2003.12.004

[9] Kidd EA, Siegel BA, Dehdashti F, Grigsby PW. The standardized uptake value for F-18 fluorodeoxyglucose is a sensitive predictive biomarker for cervical cancer treatment response and survival. Cancer. 2007;110(8):1738–44. 17786947 10.1002/cncr.22974

[10] Onal C, Reyhan M, Parlak C, Guler OC, Oymak E. Prognostic value of pretreatment 18F-fluorodeoxyglucose uptake in patients with cervical cancer treated with definitive chemoradiotherapy. Int J Gynecol Cancer. 2013;23(6):1104–10. 23792605 10.1097/IGC.0b013e3182989483

[11] Yen TC, See LC, Lai CH, Tsai CS, Chao A, Hsueh S, et al Standardized uptake value in para-aortic lymph nodes is a significant prognostic factor in patients with primary advanced squamous cervical cancer. Eur J Nucl Med Mol Imaging. 2008;35(3):493–501. 17955238 10.1007/s00259-007-0612-1

[12] Kidd EA, Siegel BA, Dehdashti F, Grigsby PW. Pelvic lymph node F-18 fluorodeoxyglucose uptake as a prognostic biomarker in newly diagnosed patients with locally advanced cervical cancer. Cancer. 2010;116(6):1469–75. 10.1002/cncr.24972 20108309

[13] Onal C, Guler OC, Reyhan M, Yapar AF. Prognostic value of ^18^F-fluorodeoxyglucose uptake in pelvic lymph nodes in patients with cervical cancer treated with definitive chemoradiotherapy. Gynecol Oncol. 2015;137(1):40–6. 10.1016/j.ygyno.2015.01.542 25641567

[14] Choi HJ, Roh JW, Seo SS, Lee S, Kim JY, Kim SK et al Comparison of the accuracy of magnetic resonance imaging and positron emission tomography/computed tomography in the presurgical detection of lymph node metastases in patients with uterine cervical carcinoma: a prospective study. Cancer. 2006;106(4):914–22. 16411226 10.1002/cncr.21641

[15] Yilmaz M, Adli M, Celen Z, Zincirkeser S, Dirier A. FDG PET-CT in cervical cancer: relationship between primary tumor FDG uptake and metastatic potential. Nucl Med Commun. 2010;31(6):526–31. 20215979 10.1097/MNM.0b013e32833800e7

[16] Roh JW, Seo SS, Lee S, Kang KW, Kim SK, Sim JS, et al Role of positron emission tomography in pretreatment lymph node staging of uterine cervical cancer: a prospective surgicopathologic correlation study. Eur J Cancer. 2005;41(14):2086–92. 16125928 10.1016/j.ejca.2005.05.013

[17] Lv K, Guo HM, Lu YJ, Wu ZX, Zhang K, Han JK. Role of 18F-FDG PET/CT in detecting pelvic lymph-node metastases in patients with early-stage uterine cervical cancer: comparison with MRI findings. Nucl Med Commun. 2014;35(12):1204–11. 25222911 10.1097/MNM.0000000000000198

[18] Grabellus F, Sheu SY, Bachmann HS, Lehmann N, Otterbach F, Heusner TA, et al The XbaI G>T polymorphism of the glucose transporter 1 gene modulates ^18^F-FDG uptake and tumor aggressiveness in breast cancer. J Nucl Med. 2010;51(8):1191–7. 10.2967/jnumed.110.075721 20679470

[19] Lee DW, Chong GO, Lee YH, Hong DG, Cho YL, Jeong SY, et al Role of SUVmax and GLUT-1 expression in determining tumor aggressiveness in patients with clinical stage I endometrioid endometrial cancer. Int J Gynecol Cancer. 2015;25(5):843–9. 25347093 10.1097/IGC.0000000000000301

[20] Nakamura K, Okumura Y, Kodama J, Hongo A, Kanazawa S, Hiramatsu Y. The predictive value of measurement of SUVmax and SCC-antigen in patients with pretreatment of primary squamous cell carcinoma of cervix. Gynecol Oncol. 2010;119(1):81–6. 10.1016/j.ygyno.2010.04.020 20580064

[21] Song BI, Lee SW, Jeong SY, Chae YS, Lee WK, Ahn BC, et al ^18^F-FDG uptake by metastatic axillary lymph nodes on pretreatment PET/CT as a prognostic factor for recurrence in patients with invasive ductal breast cancer. J Nucl Med. 2012;53(9):1337–44. 10.2967/jnumed.111.098640 22870824

[22] Groves AM, Shastry M, Rodriguez-Justo M, Malhotra A, Endozo R, Davidson T, et al ^¹⁸^F-FDG PET and biomarkers for tumour angiogenesis in early breast cancer. Eur J Nucl Med Mol Imaging. 2011;38(1):46–52. 10.1007/s00259-010-1590-2 20711577

[23] Guo J, Higashi K, Ueda Y, Oguchi M, Takegami T, Toga H, et al Microvessel density: correlation with 18F-FDG uptake and prognostic impact in lung adenocarcinomas. J Nucl Med. 2006;47(3):419–25. 16513610

[24] Schwarz JK, Siegel BA, Dehdashti F, Grigsby PW. Association of posttherapy positron emission tomography with tumor response and survival in cervical carcinoma. JAMA. 2007;298(19):2289–95. 18029833 10.1001/jama.298.19.2289

[25] Siva S, Herschtal A, Thomas JM, Bernshaw DM, Gill S, Hicks RJ, et al Impact of post-therapy positron emission tomography on prognostic stratification and surveillance after chemoradiotherapy for cervical cancer. Cancer. 2011;117(17):3981–8. 10.1002/cncr.25991 21365626

[26] Soret M, Bacharach SL, Buvat I. Partial-volume effect in PET tumor imaging. J Nucl Med. 2007;48(6):932–45. 17504879 10.2967/jnumed.106.035774

